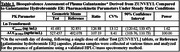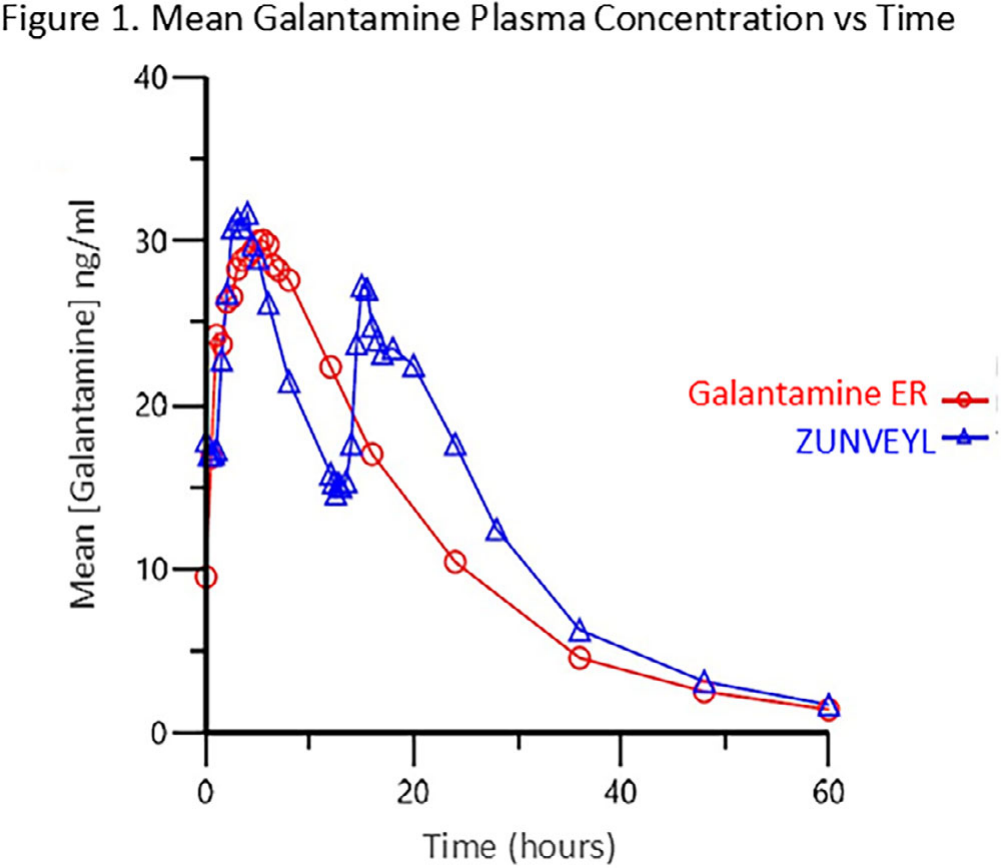# Bioequivalence of ZUNVEYL a Galantamine Prodrug to Galantamine Hydrobromide Extended‐Release Demonstrated Under Steady State Conditions

**DOI:** 10.1002/alz70859_107147

**Published:** 2025-12-26

**Authors:** Denis G Kay, Kurt P Grady, Andrew J Wahlert

**Affiliations:** ^1^ Alpha Cognition Inc, Vancouver, BC Canada

## Abstract

**Background:**

ZUNVEYL (benzgalantamine, galantamine benzoate gluconate), is a pharmacologically inactive prodrug of galantamine. ZUNVEYL was FDA approved in 2024 for BID dosing for the treatment of mild to moderate Alzheimer’s dementia, via the 505(b)(2) regulatory pathway, relying on FDA’s previous finding of safety and efficacy for the listed drugs (LDs) Razadyne® (galantamine hydrobromide tablets), and Razadyne® ER (galantamine hydrobromide extended‐release capsules). When dosed as a delayed‐release (DR) tablet, ZUNVEZYL bypasses the stomach and, is absorbed in the small intestine potentially reducing the gastrointestinal side effects common for acetylcholinesterase inhibitors. Consequently, ZUNVEYL may offer advantages over the other acetylcholinesterase inhibitors by reducing gastrointestinal adverse effects which limit patient compliance. Here we report the outcome of a study designed to assess the relative bioavailability of ZUNVEYL DR tablets to galantamine hydrobromide extended‐release (ER) capsules, under steady state conditions.

**Methods:**

This was an open‐label, balanced, randomized, multiple dose, two‐treatment, two‐arm, two‐period, comparative steady state study of ZUNVEYL DR tablets, 5 mg (BID, for 7 days) compared to galantamine hydrobromide ER, 8 mg (QD, for 7 days), in healthy adult subjects (N=40). The study protocol underwent ethics review and was conducted under GCP conditions.

**Results:**

Table 1 and Figure 1 describe the pharmacokinetic parameters calculated from the plasma analysis of galantamine derived from ZUNVEYL and galantamine hydrobromide ER. ZUNVEYL represented less than 1% of total circulating drug, which provided further evidence of safety.

**Conclusions:**

ZUNVEYL was well‐tolerated with no serious adverse event noted. The steady state study established a scientific bridge to the LD, galantamine hydrobromide ER capsules. Although the extent of absorption measured by the AUC[0‐24]ss was comparable between ZUNVEYL and the LD, the Cmaxss was slightly higher than the LD. The safety of the higher Cmax of galantamine derived from ZUNVEYL compared to galantamine hydrobromide ER capsules is supported by the lower Cmax of galantamine derived from ZUNVEYL compared to the galantamine hydrobromide immediate release tablets [see abstract # 107030].